# Evaluating physiotherapy students’ clinical reasoning skills on clients’ activity-related behaviour and behaviour change: A comparative study in South Africa using Swedish reference data

**DOI:** 10.4102/sajp.v82i1.2345

**Published:** 2026-10-06

**Authors:** Shamila Gamiet, Farhana Karachi, José Frantz, Maria Elvén, Joliana Phillips

**Affiliations:** 1 Interprofessional Education Unit, Faculty of Community and Health Sciences, University of the Western Cape, Bellville, South Africa; 2 Department of Physiotherapy, Faculty of Community and Health Sciences, University of the Western Cape, Bellville, South Africa; 3 Research, Innovation and Postgraduate Studies, Sol Plaatje University, Kimberley, South Africa; 4 Division of Physiotherapy, School of Health, Care and Welfare, Mälardalen University, Västerås, Sweden; 5 Research Development and Postgraduate Support Office, University of the Western Cape, Bellville, South Africa

**Keywords:** behavioural medicine, behaviour change, clinical reasoning, competencies, curriculum, physiotherapy education, students

## Abstract

**Background:**

Behaviour-focused clinical reasoning is essential for effective physiotherapy practice. Physiotherapy education must equip students with competencies in psychosocial and behavioural aspects of assessment and intervention. Evidence of South African physiotherapy students’ knowledge and competencies in behavioural medicine remains unexplored.

**Objectives:**

To assess behaviour-focused clinical reasoning competencies among final-year physiotherapy students at a South African university and compare these competencies with those of final-year students from two Swedish universities with integrated behavioural medicine curricula.

**Method:**

A cross-sectional comparative study design was employed. Final-year physiotherapy students (*N* = 38) from a South African university completed the Reasoning 4 Change (R4C) questionnaire. Swedish comparative data (*N* = 61) were included. Data were analysed using frequencies, percentages and independent *t*-tests. Cohen’s *d* with 95% confidence intervals (CIs) was calculated.

**Results:**

The South African cohort scored significantly lower for knowledge (mean 32.6, standard deviation [s.d.] ± 5.1 vs. 35.7 ± 4.8; *p* = 0.003; *d* = −0.63), cognition (31.7 ± 6.0 vs. 34.9 ± 5.8; *p* = 0.012; *d* = −0.53), input from client (34.2 ± 6.3 vs. 38.5 ± 7.2; *p* = 0.003; *d* = −0.62), functional behaviour analysis (21.8 ± 2.6 vs. 23.8 ± 2.9; *p* < 0.001; *d* = −0.71) and strategies for behaviour change (16.2 ± 1.9 vs. 19.6 ± 3.7; *p* < 0.001; *d* = −1.07), but scored significantly higher for self-efficacy (77.8 ± 12.5 vs. 70.4 ± 12.6; *p* = 0.005; *d* = 0.59) and contextual factors (20.2 ± 2.7 vs. 14.9 ± 4.7; *p* < 0.001; *d* = 1.31).

**Conclusion:**

Explicit behavioural medicine integration within physiotherapy education may strengthen behaviour-focused clinical reasoning competencies.

**Clinical Implications:**

Longitudinal integration of behavioural medicine content may strengthen students’ behaviour-focused clinical reasoning, client engagement and behaviour change competencies in physiotherapy practice.

## Introduction

Clinical reasoning (CR) is a foundational competency in physiotherapy and is central to safe, effective and person-centred clinical practice (Abrandt Dahlgren et al. 2022; Jones, Edwards & Jensen 2019). It involves the cognitive and metacognitive processes through which physiotherapists gather, interpret and integrate information; generate and test hypotheses; make decisions; and reflect on outcomes in collaboration with clients (Furze et al. 2015; Higgs & Jensen 2019). Clinical reasoning is therefore more than technical problem-solving; it is a complex, situated and iterative process shaped by professional knowledge, contextual factors and the client’s lived experience and expectations (Young et al. 2020). World Physiotherapy highlights the need for physiotherapists to develop CR skills that demonstrate a holistic perspective, reflective capacity and meaningful engagement with the clients’ goals to support optimal health (World Physiotherapy 2022).

The increasing global burden of non-communicable diseases (NCDs), chronic pain, persistent disability and lifestyle-related conditions necessitates a broader understanding of human behaviour and psychosocial influences on health (Hassett et al. 2022; Vos et al. 2020).

Contemporary physiotherapy practice demands competencies that integrate behavioural science, health psychology and psychosocial frameworks to support behaviour change, adherence, self-management and long-term health maintenance (Fritz & Overmeer 2023; Gervais-Hupé et al. 2022; Holopainen et al. 2020).

Despite the recognised importance of these competencies, research consistently shows that physiotherapists and physiotherapy students often struggle to incorporate psychosocial and behavioural dimensions in assessment and intervention planning (Alexanders, Anderson & Henderson 2015; Driver et al. 2017; Fritz & Overmeer 2023). This gap underscores the importance of exploring how educational programmes prepare students for behaviour-focused CR.

Behaviour-focused CR is particularly relevant because movement, function and activity patterns can be understood as behaviours shaped by biological, psychological and social factors (Elvén et al. 2018a). Target behaviours, such as avoidance as a result of pain, sedentary routines or limited engagement in exercise, serve as key determinants of clinical outcomes.

Effective physiotherapy therefore requires not only identifying physical impairments but also analysing behaviours that contribute to disability and implementing evidence-based strategies to support healthier patterns of activity (Gervais-Hupé et al. 2022; Linton & Shaw 2011).

Efforts to strengthen behaviour-focused CR have led to the integration of behavioural medicine content and competencies (BMCC) into physiotherapy curricula in several countries, notably Sweden, over the past two decades (Elvén et al. 2015; Sandborgh et al. 2020). Behavioural medicine in physiotherapy draws on a biopsychosocial model and incorporates learning theory, cognitive behavioural frameworks, motivational interviewing and behavioural analysis to enhance treatment planning and therapeutic effectiveness (Holopainen et al. 2020; Norris et al. 2019; Sandborgh et al. 2020). Curricula aligned with these principles aim to support students in understanding how behavioural patterns interact with environmental and psychosocial determinants and how this understanding can be applied within CR (Elvén et al. 2015).

A key framework for operationalising these competencies is the Clinical Reasoning Focused on Behaviour Change for Physiotherapists (CRBC-PT) model developed by Elvén et al. (2015).

This model integrates traditional physiotherapy reasoning with behaviour change theories and emphasises five interconnected components: The physiotherapist, the client, input from the client, functional behavioural analysis and behaviour change strategies. The model positions CR as a shared process, wherein physiotherapists and clients work collaboratively to identify activity-related target behaviours, analyse the biopsychosocial factors influencing these behaviours, and implement strategies for change. Competent performance within this framework requires strong knowledge, analytical reasoning, self-efficacy, reflective skills and the ability to meaningfully engage clients throughout the reasoning and decision-making process.

This model provides a theoretical foundation for understanding how students develop competencies in behaviour-focused CR (Elvén et al. 2015).

Despite the growing body of research on behaviourally informed physiotherapy education in Europe, North America and Australia (Cabebe et al. 2023; Hassett et al. 2022; Norris et al. 2019), limited evidence exists regarding South African physiotherapy students’ competencies in this area. South Africa faces a substantial burden of NCDs, chronic pain conditions and disability, making behaviour change interventions essential for improving health outcomes (Mayosi et al. 2012; Naidoo et al. 2018). This context amplifies the need for physiotherapists who can integrate psychosocial and behavioural considerations into practice.

South African research emphasises that psychosocial factors are often stronger predictors of pain persistence and disability than physical impairments and calls for physiotherapists to develop stronger behaviour change competencies (Diener 2021; Kahere & Ginindza 2022). Physiotherapy training in South Africa has been progressively aligned with holistic, community-based and person-centred principles, consistent with National Health Insurance and primary health care reforms (Shumba & Tekian 2024).

However, empirical studies indicate that curricula remain predominantly biomedical with inconsistent incorporation of behavioural medicine content (Driver et al. 2017).

Consequently, the extent to which South African students develop competencies aligned with behaviourally focused CR remains unclear. This gap is particularly concerning given evidence that curriculum content directly influences students’ development of CR and professional self-efficacy. Understanding how South African students’ competencies compared to those trained in BMCC-enriched programmes may offer valuable insights for strengthening local curricula. Although our study compares students from South Africa and Sweden, the findings should not be interpreted as representative of all physiotherapy programmes within either country. Rather, our study compares one South African programme with two Swedish programmes that explicitly integrated BMCC into their curricula. The comparison was intended to explore how differing curricular emphases may relate to students’ behaviour-focused CR competencies.

Despite increased recognition of the need for bio-psychosocially informed physiotherapy practice in South Africa, there is no empirical baseline describing physiotherapy students’ competencies in behaviour-focused CR. Establishing such a baseline is critical for informing curriculum evaluation and transformation efforts across the country. Understanding where students currently stand provides a foundation for benchmarking local programmes against international standards.

This baseline is relevant in the context of South Africa’s current health system reforms. The imminent implementation of the National Health Insurance emphasises person-centred care in which physiotherapists play a significant role in facilitating health behaviour change, chronic disease management and self-management strategies (Mudzi 2025).

Identifying competency gaps in behaviour-focused CR is not only a pedagogical concern but also a strategic national priority for preparing a workforce capable of meeting National Health Insurance’s goals. By comparing students from a South African physiotherapy programme with peers from two Swedish programmes where BMCC are explicitly embedded in physiotherapy curricula, the present study offers empirical insights that can guide national curriculum transformation. The findings may assist this particular South African higher education institution in identifying specific domains, such as functional behavioural analysis, client-centred input and behaviour change strategies, that require targeted strengthening. Such evidence is vital for aligning curriculum with global competency frameworks, improving graduate readiness for evolving clinical demands, and ensuring that physiotherapy education supports national health priorities. Ultimately, our study contributes to a clearer understanding of how a local programme can evolve to integrate behavioural medicine more intentionally and effectively.

The Reasoning 4 Change (R4C) questionnaire, developed to evaluate students’ and clinicians’ CR skills related to behaviour change, provides a validated means of assessing these competencies across domains such as knowledge, cognition, metacognition, client input, behavioural analysis and strategies for behaviour change (Elvén et al. 2015). Previous studies using the R4C instrument in Swedish cohorts suggest that students who receive structured behavioural medicine education show stronger reasoning patterns and higher self-efficacy for behaviour change compared with those studying in traditional programmes (Elvén, Dean & Söderlund 2021).

Given the absence of research on behaviour-focused CR within South African physiotherapy training, our study addresses a critical gap. Comparing students from a single South African university with students from two Swedish universities who have received formal behavioural medicine education, can provide insight into the influence of curricular content on students’ reasoning competencies and inform curriculum development within the South African context. Accordingly, the aims of our study were: (1) to assess final-year physiotherapy students’ CR skills related to activity-related behaviour and behaviour change at a South African university, and (2) to compare these skills with those of final-year students enrolled at two Swedish universities that integrate BMCC.

## Research methods and design

### Research design

Our study employed a quantitative approach with a cross-sectional retrospective comparative design.

### Research setting

Our study was conducted at the University of the Western Cape (UWC) in South Africa, where a 4-year programme, leading to a Bachelor of Science in Physiotherapy, is offered. Graduates must complete a compulsory 1-year community service, after which they can apply to work in any private or public setting. The duration of the Swedish physiotherapy education is 3 years, leading to a Bachelor of Science Degree in Physiotherapy. After graduation, students can apply for a license to practice physiotherapy. Comparative data were obtained from students attending two physiotherapy programmes in Sweden that were purposefully selected because they explicitly incorporated BMCC within their curricula, as described in previous studies (Elvén et al. 2015, 2021). The Swedish dataset was obtained from Maria Elvén, who conducted the research in Sweden. Our study therefore consisted of a previously validated, historical dataset collected under ethical approval in Sweden, which serves as an established benchmark. The South African institution was selected as a case example within the local context and was not intended to represent all South African physiotherapy programmes.

### Population and sampling

Our study population included all final-year physiotherapy students enrolled at UWC in South Africa (*N* = 49 eligible; *n* = 38 participated) and retrospective data of final-year students from two Swedish universities (*N* = 98 eligible; *n* = 61 participated). All eligible South African students were invited to participate; therefore, our study employed a voluntary response convenience sampling approach. Participation depended on voluntary consent and completion of the questionnaire. The South African cohort yielded a response rate of 77.5%, while the Swedish cohort yielded a response rate of 62%.

### The Reasoning 4 Change instrument

The R4C instrument, developed by Elvén et al. (2018a, 2018b), was used to assess the CR of South African and Swedish students. The instrument includes four domains: domain 1 (D1) = the physiotherapist, domain 2 (D2) = input from client, domain 3 (D3) = functional behavioural analysis and domain 4 (D4) = strategies for behaviour change. The first domain includes six subscales: D1.1 knowledge, D1.2 cognition, D1.3 metacognition, D1.4a attitudes, D1.4b self-efficacy and D1.5 contextual factors according to their CR. Subscale D1.4 refers to psychological factors and consists of two components namely; attitudes and self-efficacy. Domains 2, 3 and 4 are built up of case scenarios starting with a minimum of information and then gradually extended with new information. The subscales of D1 consist of students’ self-ratings of statements. The scoring key of the R4C instrument is based on the distribution of answers provided by physiotherapists with expertise in physiotherapy with a behavioural medicine approach. Higher scores on the R4C domains indicate stronger behaviour-focused CR competencies. A detailed description of the instrument has been published previously (Elvén et al. 2018a, 2018b).

The R4C instrument in Swedish has demonstrated excellent content validity based on relevance ratings by physiotherapy experts and satisfactory psychometric properties including internal consistency, test-retest reliability, and construct validity (Elvén, Boersma & Overmeer 2022; Elvén et al. 2018b). The R4C instrument was translated into English using a rigorous process with forward and back translation conducted by two professional translators and input from physiotherapy practitioners and researchers with Swedish and English (South African) as their mother tongue.

### Data collection

Final-year physiotherapy students from UWC were invited via email to participate. The primary author (S.G.) met with students in person to provide detailed instructions. Hard copies of the R4C were given to consenting students to complete at home, in their own time.

The primary author (S.G.) was available either in person or telephonically for students who had any questions. Data from the Swedish cohort were collected in a previous study (Elvén et al. 2019). The Swedish version of the instrument was a web-based format, and the students answered the instrument on computers in a lecture hall at the university. Although both cohorts completed the same validated R4C instrument, differences in administration procedures existed. Students from the South African physiotherapy programme completed paper-based questionnaires independently at home, whereas students from the two Swedish programmes completed a supervised web-based version in a university setting. These differences were considered during interpretation of findings.

### Data analysis

The data were analysed using the Statistical Package for Social Sciences (SPSS) version 29.0. Descriptive statistics for demographic variables and scores for the four domains of the R4C instrument were calculated using frequencies, means and standard deviations. Independent *t*-tests were used to compare scores between the South African and Swedish cohorts. Results were considered statistically significant at *p* < 0.05. Cohen’s d effect sizes and 95% confidence intervals (CIs) were calculated for all between-group comparisons to determine the magnitude, precision and practical significance of findings. Effect sizes were interpreted as small (0.2), medium (0.5) and large (0.8).

### Ethical considerations

An application for full ethical approval was made to the UWC Humanities and Social Science Research Ethics Committee, and ethics consent was received on 16 October 2025. The ethics approval number is HSSREC Reference Number: HS18/8/10. Participants received information sheets outlining the study’s purpose and procedures. Written informed consent was obtained from all participants. Participants were informed that their participation was entirely voluntary, with no impact on their academic status or professional relationships, and that they could withdraw at any time without consequence. For the research conducted in Sweden, ethical approval was obtained from the Regional Ethical Review Board, Uppsala, Sweden (dnr 2013/020), and written informed consent was obtained from participants in the original study. The Swedish dataset was anonymised before being shared with the research team. No identifiable participant information was accessible to the authors. Data from both countries were securely stored on password-protected institutional devices accessible only to the research team and managed according to institutional ethical guidelines.

## Results

### Demographic profile of the participants

In total, 38 final-year physiotherapy students from UWC participated, corresponding to a response rate of 77.5%. The mean age of participants was 23 (standard deviation [s.d.]: ± 1.37; range: 21–25) years. Most participants were female (71%, *n* = 27), with males comprising 29% (*n* = 11). From the secondary data obtained from Sweden, 61 final-year physiotherapy students participated, corresponding to a response rate of 62%. Their mean age was 25 (s.d.: ± 3.7; range: 22–41) years, with 72% (*n* = 44) female participants (Table 1).

**TABLE 1:** Demographic profile of participants.

Characteristics	UWC students from the South African physiotherapy programme (*N* = 38)				Students from the two Swedish physiotherapy programmes (*N* = 61)			
	*n*	%	± s.d.	Range	*n*	%	± s.d.	Range
**Gender**								
Male	11	29	-	-	17	28	-	-
Female	27	71	-	-	44	72	-	-
**Age categories (years)**								
21–24	29	76	-	-	36	59	-	-
25–28	9	24	-	-	22	36	-	-
30–34	-	-	-	-	1	2	-	-
35–41	-	-	-	-	2	3	-	-
**Age**	-	-	± 1.37	21–25	-	-	± 3.7	22–41
Years	23	-	-	-	25	-	-	-

UWC, University of the Western Cape; s.d., standard deviation.

### Reasoning 4 Change outcomes

The final-year physiotherapy students from the South African university scored significantly lower than students from the two Swedish universities in knowledge (32.6 ± 5.1 vs. 35.7 ± 4.8; *p* = 0.003; *d* = −0.63, 95% CI [−1.05 to −0.22]), cognition (31.7 ± 6.0 vs. 34.9 ± 5.8; *p* = 0.012; *d* = −0.53, 95% CI [−0.94 to −0.12]), input from client (34.2 ± 6.3 vs. 38.5 ± 7.2; *p* = 0.003; *d* = −0.62, 95% CI [−1.03 to −0.21]), functional behaviour analysis (21.8 ± 2.6 vs. 23.8 ± 2.9; *p* < 0.001; *d* = −0.71, 95% CI [−1.13 to −0.29]) and strategies for behaviour change (16.2 ± 1.9 vs. 19.6 ± 3.7; *p* < 0.001; *d* = −1.07, 95% CI [−1.49 to −0.63]). These findings reflected medium to large effect sizes, with the largest difference observed for strategies for behaviour change.

The South African cohort scored significantly higher than the Swedish cohort in self-efficacy in CR focused on behaviour change (77.8 ± 12.5 vs. 70.4 ± 12.6; *p* = 0.005; *d* = 0.59, 95% CI [0.17–1.00]) and contextual factors (20.2 ± 2.7 vs. 14.9 ± 4.7; *p* < 0.001; *d* = 1.31, 95% CI [0.86–1.75]), representing medium and large effect sizes, respectively.

No significant differences were found for metacognition or attitudes towards behaviour-focused CR, both of which demonstrated small effect sizes with CIs crossing zero (Table 2).

**TABLE 2:** Comparison of the scores of two groups of students (Reasoning 4 Change instrument).

Outcomes	UWC students from the South African physiotherapy programme not receiving BMCC (*N* = 38)		Students from the two Swedish physiotherapy programmes receiving BMCC (*N* = 61)		Between-group difference (*t*-test) *p*-value	Cohen’s *d* effect size	95% CI for Cohen’s *d*	Interpretation
	Mean	± s.d.	Mean	± s.d.				
**R4C Instrument: Domain and subscales**								
D1.1 Physiotherapy students: knowledge	32.6	± 5.1	35.7	± 4.8	0.003	−0.63	−1.05 to −0.22	Medium
D1.2 Physiotherapy students: cognition	31.7	± 6.0	34.9	± 5.8	0.012	−0.53	−0.94 to −0.12	Medium
D1.3 Physiotherapy students: metacognition	39.0	± 5.5	37.4	± 5.8	0.184	0.28	−0.13 to 0.68	Small
D1.4 Physiotherapy students: attitudes towards CR focused on behaviour change	83.8	± 11.0	80.5	± 10.4	0.137	0.31	−0.1 to 0.72	Small
D1.4 Physiotherapy students: self-efficacy in CR focused on behaviour change	77.8	± 12.5	70.4	± 12.6	0.005	0.59	0.17 to 1.00	Medium
D1.5 Physiotherapy students: contextual factors	20.2	± 2.7	14.9	± 4.7	< 0.001	1.31	0.86 to 1.75	Large
D2 Input from client	34.2	± 6.3	38.5	± 7.2	0.003	−0.62	−1.03 to −0.21	Medium
D3 Functional behaviour analysis	21.8	± 2.6	23.8	± 2.9	< 0.001	−0.71	−1.13 to −0.29	Medium to Large
D4 strategies for behaviour change	16.2	± 1.9	19.6	± 3.7	< 0.001	−1.07	1.49 to −0.63	Large

Note: The theoretical minimum and maximum scores for the Reasoning 4 Change instrument were: D1.1 knowledge = 8–48, D1.2 cognition = 7–46, D1.3 metacognition = 8–48, D1.4 attitudes = 0–100, D1.4 self-efficacy = 0–100, D1.5 contextual factors = 5–30, D2 input from client = 0.6–66.1, D3 functional behavioural analysis = 3.8–34.3, D4 strategies for behaviour change = 0–36.4. Higher scores reflect stronger behaviour-focused CR competencies according to the scoring framework of the R4C instrument.

R4C, Reasoning 4 Change; CR, clinical reasoning; UWC, University of the Western Cape; BMCC, behavioural medicine content and competencies; CI, confidence interval; s.d., standard deviation.

## Discussion

Our study demonstrates differences in behaviour-focused CR between students from one South African and two Swedish physiotherapy programmes and provides insight into how educational and contextual factors, including curriculum design, may influence the development of these competencies. Students from the two Swedish universities performed significantly better in the key domains of behaviour-focused CR, namely: knowledge, cognition, client input, functional behavioural analysis and strategies for behaviour change, which aligns with the theoretical foundation outlined in the introduction, particularly the CRBC-PT model and the emphasis on BMCC. These findings align with previous research demonstrating that curricula integrating BMCC produce students with stronger behavioural reasoning skills (Elvén et al. 2021) and suggest that explicit behavioural medicine curriculum exposure may contribute to stronger behaviour-focused CR competencies and support consideration of enhanced behavioural medicine integration within South African physiotherapy curricula.

Although the findings align with differences in behavioural medicine curriculum exposure, curriculum content alone is unlikely to fully explain the observed performance differences. Other factors, including educator expertise, pedagogical approaches, assessment culture, quality of clinical supervision, institutional priorities and broader educational contexts, may also have influenced students’ competencies.

### Behaviour-focused competencies: Interpreting the performance gaps

The superior performance of the Swedish cohort across key R4C domains may partly reflect the influence of structured behavioural medicine training, which has been associated with stronger behavioural CR in previous studies (Elvén et al. 2021). However, these differences are unlikely to be explained by curriculum content alone and are more plausibly shaped by variation in educator expertise, assessment practices, clinical supervision quality and student demographic characteristics across institutions. In contrast, the lower performance of the South African cohort in key R4C domains such as knowledge, client input, functional behavioural analysis and behaviour change strategies reflects the more limited behavioural medicine coverage in the local curriculum. These findings may suggest that limited exposure to explicit behavioural frameworks within training may constrain students’ ability to systematically identify activity-related target behaviours, analyse determinants of behaviour and design evidence-based strategies, which are all critical components of the CRBC-PT model (Elvén et al. 2015). Importantly, the observed differences were not only statistically significant but also demonstrated medium to large effect sizes, particularly for functional behavioural analysis, contextual factors and strategies for behaviour change. This suggests that the differences between cohorts may have meaningful educational and practical relevance rather than reflecting small statistical variations alone.

The reduced knowledge and cognition scores among students from the South African physiotherapy programme are also consistent with the emphasis on the need for a biopsychosocial and behavioural perspective in modern physiotherapy (Driver et al. 2017; Hassett et al. 2022; Norris et al. 2019). Without this foundation, students may struggle to integrate client-reported information, contextual influences and behavioural determinants into the reasoning process, limiting their ability to deliver holistic, person-centred care.

The performance gaps observed between the South African and Swedish cohort align with well-documented curriculum characteristics within South African physiotherapy education. Despite national policy shifts emphasising holistic, person-centred care, South African physiotherapy curricula remain largely influenced by a biomedical orientation, with inconsistent integration of behavioural medicine, health psychology and psychosocial skill development (Shumba & Tekian 2024). Studies have shown that South African programmes typically emphasise anatomy, pathology and traditional impairment-focused CR, while psychosocial assessment, motivational interviewing, functional behavioural analysis and behaviour change techniques receive limited structured coverage or are embedded inconsistently across modules (Unger et al. 2025). This curricular pattern may have contributed to lower scores in the domains of client input, functional behavioural analysis and behaviour change strategies – competencies known to require explicit instruction and repeated guided practice. International literature consistently suggests that without formal behavioural medicine content, students struggle to apply biopsychosocial frameworks in CR, despite recognising their importance (Elvén et al. 2021; Holopainen et al. 2020). Furthermore, the collective literature on CR education emphasises learning activities in progression throughout the curriculum (Elvén et al. 2023).

These findings underscore the need for intentional curricular redesign in South Africa to embed behavioural competencies longitudinally and systematically, rather than relying on ad hoc exposure during clinical placements. Interpretation of these findings should also consider broader systemic differences between the educational contexts. The South African and Swedish programmes differ in programme duration, entry pathways, clinical exposure structures and healthcare systems, all of which may independently shape students’ CR competencies and perceptions of practice readiness. South African physiotherapy education typically spans 4 years and includes extensive community-based and primary healthcare exposure within resource-constrained environments, whereas Swedish physiotherapy education is generally a 3-year programme with different educational pathways and healthcare structures. These systemic differences may influence students’ CR development, confidence, contextual awareness and opportunities for behaviour-focused practice independently of behavioural medicine curriculum content.

Consequently, the observed differences between cohorts should be interpreted as reflecting multiple interacting educational and contextual influences rather than being attributable solely to behavioural medicine curriculum content.

### Self-efficacy and perceived contextual support

Although the students from the South African university reported high perceived self-efficacy in behavioural reasoning skills, their actual knowledge in this domain was limited. Recent studies similarly suggest that students frequently overestimate their performance or self-efficacy relative to objective measures of skill and knowledge (Gabbard & Romanelli 2021; Knof, Berndt & Shiozawa 2024). In contrast, Elvén et al. (2021) found that students who received BMCC training typically reported higher confidence and stronger behaviour-focused CR, highlighting a divergence from expected patterns.

Understanding this divergence has important implications for curriculum design and student preparedness.

One explanation relates to the UWC physiotherapy programme, where students engage in extensive primary healthcare rotations and interprofessional activities throughout the 4-year curriculum. These experiences may enhance general confidence in communication, client engagement and collaborative practice (Furze et al. 2015; Nørgaard et al. 2013; Norman et al. 2017), even without explicit training in behaviour change theories or techniques. The mismatch between perceived and actual competence suggests overestimation of ability or insufficient calibration of self-efficacy (Gabbard & Romanelli 2021; Knof et al. 2024). Without structured opportunities to apply behaviour change principles, receive targeted feedback or be assessed against explicit behavioural medicine criteria, students may rely on general communication skills as a proxy for behaviour change competence. This can create confidence that is not grounded in specialised frameworks or behaviour change techniques.

Broader literature supports this interpretation. Students trained primarily in biomedical models may develop confidence through repeated hands-on exposure, even when specific behavioural skills are not taught (Furze et al. 2015; Norman et al. 2017). This can inadvertently encourage overestimation in complex domains like behaviour change, which require both theoretical understanding and deliberate skill acquisition rather than intuitive communication alone.

This finding highlights a critical educational concern: Students must not only feel confident but also acquire targeted competencies to facilitate behaviour change effectively. Confidence without competence may limit physiotherapists’ ability to effectively support behaviour change in clinical practice (Knof et al. 2024). The discrepancy between self-rated efficacy and objectively measured performance underscores the need for curricula that intentionally develop and assess both confidence and competence, embedding structured behavioural medicine teaching, deliberate practice and tools to support accurate self-appraisal.

### Attitudes and contextual factors

Both student groups held similarly positive attitudes toward biopsychosocial practice, with no significant differences observed. This mirrors findings reported by Elvén et al. (2021) and suggests that positive attitudes alone are insufficient to translate into competent behaviour-focused reasoning without supporting curricular content and practical experience. Students across both contexts valued holistic, person-centred care and recognised the importance of psychosocial factors, yet only those with structured BMCC training demonstrated the knowledge and skills to operationalise these values in practice.

The significantly higher contextual factor scores observed in the South African cohort may reflect their substantial exposure to community-based education, social accountability initiatives and primary healthcare settings, which are hallmarks of South African physiotherapy training (Shumba & Tekian 2024; Unger et al. 2025). These environments increase awareness of social determinants of health, resource constraints and contextual barriers to patient adherence, which may influence students’ reasoning even in the absence of formal behavioural medicine training. In contrast, Swedish students’ programmes emphasise structured behavioural competencies and cognitive frameworks but may offer fewer real-world community exposures. Thus, the combination of contextual immersion and general therapeutic confidence, rather than competency in behavioural analysis, may explain the South African students’ elevated self-efficacy and contextual reasoning scores.

While contextual awareness is valuable, it does not substitute for specific competencies in behaviour analysis. As Elvén et al. (2021) observed, students from traditional curricula often rate contextual support highly while demonstrating weaker behaviour change skills. These findings highlight the need to consider broader educational context when addressing competency gaps and designing curricula that foster both positive attitudes and the practical skills required for behaviour-focused reasoning.

### Implications for the South African context

These findings have important implications for physiotherapy education in South Africa. As the country faces persistent health inequities and a quadruple burden of disease, including high prevalence of chronic NCDs and disability, physiotherapists must be prepared to work beyond a purely biomedical model (Unger et al. 2025). Addressing these challenges requires physiotherapists who understand patient behaviour, motivation and the social determinants of health – competencies that extend beyond traditional physical assessment and treatment.

The SASP and recent reviews emphasise that psychosocial factors are powerful predictors of outcomes and call for competency-based education that includes behaviour change skills (Diener 2021; Kahere & Ginindza 2022). In line with global competency trends and the World Physiotherapy framework, curricula must explicitly prepare graduates for these broader roles (Shumba & Tekian 2024; World Physiotherapy 2022). Our study suggests that without purposeful curricular reform, South African students may graduate underprepared in this critical area of practice.

The biopsychosocial model, which underpins contemporary physiotherapy practice, requires more than theoretical acknowledgement – it demands practical competencies in behavioural assessment and intervention.

Our findings suggest that the curriculum at one South African university has successfully fostered positive attitudes towards holistic care but has not yet developed the structured educational pathways needed to build corresponding competencies. This gap between values and capabilities must be addressed through intentional curriculum redesign.

### Recommendations for curriculum enhancement

Building on the gaps identified and international best practice, several curriculum recommendations emerge:

- Integrate behavioural medicine content longitudinally, ensuring that behaviour change theory, communication skills, functional behavioural analysis and intervention strategies are introduced early and reinforced across modules.
- Embed structured behavioural reasoning frameworks, such as the CRBC-PT model, to scaffold student thinking and move learners beyond intuition towards systematic and reflective decision-making.
- Increase authentic, supervised opportunities for behaviour change practice, particularly within primary health care and community settings, allowing students to apply behavioural strategies in real-world contexts.
- Strengthen assessment of behavioural CR, incorporating tools like the R4C instrument to monitor student development and ensure alignment between perceived and actual competence.
- Support faculty development, enabling lecturers to confidently teach behavioural frameworks, model reasoning processes and provide targeted feedback on students’ behavioural reasoning skills.

While these recommendations may inform curriculum development, the findings should be interpreted as exploratory and context-specific rather than representative of all physiotherapy programmes in South Africa or Sweden.

### Study limitations

While these findings offer important insights, several limitations warrant consideration. The cross-sectional design precludes causal inferences regarding the relationship between curriculum exposure and behaviour-focused CR competencies. Furthermore, our study included students from only one South African and two Swedish physiotherapy programmes, and a comprehensive review of physiotherapy curricula across all institutions in either country was beyond the scope of our study. Given the limited institutional sample, the findings may not fully reflect behaviour-focused CR competencies across all physiotherapy programmes in South Africa or Sweden.

Important systemic differences also existed between the educational contexts, such as programme duration, entry requirements, clinical exposure, assessment practices and healthcare systems. These factors may have influenced student performance independently of behavioural medicine curriculum exposure. The study did not directly evaluate curriculum content, teaching quality or educator competencies within participating programmes.

The Swedish dataset was obtained from a previously conducted study rather than collected contemporaneously with the South African cohort. Changes in curricula, educational practices, student characteristics or healthcare contexts over time may therefore have affected comparability between cohorts.

Administration conditions differed substantially between groups. Students from the South African university completed paper-based questionnaires independently at home, whereas students from the two Swedish universities completed a supervised web-based version in a university setting. These differences may have influenced engagement, response effort, fatigue or opportunities to consult external resources, potentially introducing measurement bias.

Although the R4C instrument demonstrates acceptable psychometric properties, written case scenarios and self-report measures cannot fully capture behaviour-focused CR during authentic clinical encounters. Cultural differences in self-assessment tendencies and confidence reporting may have influenced responses, particularly within self-efficacy domains.

## Conclusion

Students from the two Swedish physiotherapy programmes with explicit behavioural medicine curriculum content demonstrated stronger performance across several domains of behaviour-focused CR, particularly in knowledge application, cognitive reasoning, incorporation of client input, functional behavioural analysis and selection of behaviour change strategies. In contrast, students from the South African university demonstrated higher self-efficacy and contextual awareness despite lower objective performance in several behavioural reasoning domains.

These findings suggest that positive attitudes and confidence alone may be insufficient to support competent behaviour-focused CR without explicit curricular training and structured opportunities for skill development.

The findings support consideration of more explicit and longitudinal integration of behavioural medicine competencies within physiotherapy curricula to strengthen students’ ability to apply psychosocial and behaviour change principles through structured reasoning frameworks and authentic learning experiences. Such approaches may better prepare graduates for person-centred physiotherapy practice within complex healthcare settings.
